# Designs for adding a treatment arm to an ongoing clinical trial

**DOI:** 10.1186/s13063-020-4073-1

**Published:** 2020-03-06

**Authors:** Maxine Bennett, Adrian P. Mander

**Affiliations:** MRC Biostatistics Unit, University of Cambridge, Cambridge, UK

**Keywords:** Adding a treatment arm, Adaptive design, Multiple testing, Family-wise error rate control, Optimal allocation

## Abstract

**Background:**

For many disease areas, there are often treatments in different stages of the development process. We consider the design of a two-arm parallel group trial where it is planned to add a new experimental treatment arm during the trial. This could potentially save money, patients, time and resources; however, the addition of a treatment arm creates a multiple comparison problem. Current practice in trials when a new treatment arm has been added is to compare the new treatment only to controls randomised concurrently, and this is the setting we consider here. Furthermore, for standard multi-arm trials, optimal allocation randomises a larger number of patients to the control arm than to each experimental treatment arm.

**Methods:**

In this paper we propose an adaptive design, the aim of which is to adapt the sample size of the trial when the new treatment arm is added to control the family-wise error rate (FWER) in the strong sense, whilst maintaining the marginal power of each treatment-to-control comparison at the level of the original study. We explore optimal allocation for designs where a treatment arm is added with the aim of increasing the overall power of the study, where we define the overall power to be the probability of detecting all treatments that are better than the control.

**Results and conclusions:**

An increase in sample size is required to maintain the marginal power for each pairwise comparison when adding a treatment arm if control of the FWER is required at the level of the type I error in the original study. When control of the FWER is required in a single trial which adds an additional experimental treatment arm, but control of the FWER is not required in separate trials, depending on the design characteristics, it may be better to run a separate trial for each experimental treatment, in terms of the number of patients required. An increase in overall power can be achieved when optimal allocation is used once a treatment arm has been added to the trial, rather than continuing with equal allocation to all treatment arms.

## Background

Often in clinical trials different experimental drugs are compared to the same control treatment in separate trials. Having multiple experimental treatments and a single control treatment in a clinical trial can therefore save time, money and resources [1]. In this paper we explore how to design a trial where it is pre-planned to add a new experimental treatment arm part way through the trial. It is assumed that adding the new experimental treatment arm is pre-planned, but the time that the new treatment arm is added does not have to be pre-specified. Some aspects of when a new treatment arm is added during an ongoing trial are considered in the “Comparison of a single trial adding a treatment arm to independent trials” section. The methods proposed are likely to be most suited to phase III trials, since they focus on multiplicity adjustments and controlling the family-wise error rate (FWER); however, they may also be considered for phase II trials. There are multiple reasons for adding a new treatment arm to an ongoing study, such as a treatment about to complete phase II development in the same disease area or a treatment currently awaiting regulatory approval. There are administrative advantages of adding a treatment arm to an already up and running and established trial, such as the case of one protocol, with new treatments incorporated as an amendment and utilising the existing trial infrastructure (e.g. staff, protocols, recruitment, randomisation). Statistically, having one control arm for multiple experimental treatments may result in a trial that requires fewer patients than running a separate trial for each experimental treatment versus control. One of the main factors to consider when adding a treatment arm to an ongoing trial is that we may create a multiple comparison problem (if an experimental treatment arm is added to an ongoing trial with two arms comparing experimental to control) or increase the number of comparisons of treatment to control in a trial, requiring multiplicity adjustments to be made.

Our main aims for the design of a trial where a treatment arm is added are:

- To control the FWER for multiple comparisons of treatment to control
- To maintain or increase the power of each pairwise comparison of an experimental treatment to control
- To determine the optimal allocation to each treatment arm after a new treatment arm is added.

In the “Dunnett test” section we describe the method proposed by Dunnett [2] for comparing multiple experimental treatments to control, and in the “Extension of the Dunnett test when a treatment arm is added during a trial: determining the correlation between test statistics” section we extend the original [2] correlation to handle adding a treatment arm during the trial. A design is proposed in the “Adaptive design” section that adapts the sample size of all treatment arms when a new treatment arm is added to control the FWER for a specific marginal power for each treatment-to-control comparison. Optimal allocation to each treatment group is then explored for a fixed total sample size in the “Optimal allocation” section. The methodology is illustrated with an example in the “Results” section.

## Methods

### Notation

Let $j= 0,\dots,J$ represent the treatment group, where *j*=0 represents the control group and there are *J* experimental treatment groups. Let $k =1,\dots,K$ denote the stages of the trial. Let *i* represent the patients within each stage and treatment group, $i=1,\dots,n_{jk}$. *n*_*jk*_ denotes the number of patients in treatment group *j* in stage *k*. Let *μ*_*j*_ denote the true population mean for treatment *j* and *σ* denote the common standard deviation for all treatment groups, which is assumed to be known. Let *Δ*_*j*_=*μ*_*j*_−*μ*_0_.

Let *X*_*ijk*_ denote the outcome for patient *i* on treatment *j* in stage *k*. Let $\bar {X}_{jk}$ denote the sample mean of treatment *j* in stage *k*. Furthermore, when considering the control arm, let *k*_(*j*)_ denote the set of stages for which controls are randomised concurrently to treatment *j*. Let $\bar {X}_{j}$ and *n*_*j*_ denote the sample mean and sample size of treatment group *j* incorporating data from all *K* stages of the trial.

It is assumed that *X*_*ijk*_ is independent and identically distributed (iid) *N*(*μ*_*j*_,*σ*^2^). It is also assumed that the patient population is homogeneous across stages; this is also the primary assumption for sequential test procedures and for platform trials [3, 4]. The methods presented in this paper make probability statements about the true underlying treatment differences.

### Dunnett test

In this section we are considering the case of *K*=1, which corresponds to a single-stage design. The test statistic comparing each experimental treatment *j* (*j*=1,...,*J*) to control is given by [2]

1 $$  U_{j} = \frac{\bar{X}_{j}-\bar{X}_{0}}{\sqrt{\frac{1}{n_{j}}+\frac{1}{n_{0}}}}.  $$

Under the null hypothesis of no treatment difference, $(U_{1},\dots,U_{J})^{\prime }\sim MVN(\boldsymbol {0},\Sigma _{U})$, where ***0*** is the zero vector of length *J*, *Σ*_*U*_ is the *J*×*J* matrix with *jj*^*th*^ (*j*=1,...,*J*) entry equal to *σ*^2^ and *ij*^*th*^ (*i*≠*j*,*i*=1,...,*J*,*j*=1,...,*J*) entry equal to *σ*^2^*ρ*_*i*,*j*_, and correlation

2 $$  \rho_{i, j} = 1\left/ \sqrt{ \left(\frac{n_{0}}{n_{i}} + 1\right) \left(\frac{n_{0}}{n_{j}} + 1\right) }\right..  $$

Considering the standardised test statistics, *Z*_*j*_=*U*_*j*_/*σ*, under the null hypothesis, $(Z_{1},\dots,Z_{J})^{\prime }\sim MVN(\boldsymbol {0},\Sigma _{Z})$, where ***0*** is the zero vector of length *J*, *Σ*_*Z*_ is the *J*×*J* matrix with *jj*^*th*^ (*j*=1,...,*J*) entry equal to 1 and *ij*^*th*^ (*i*≠*j*,*i*=1,...,*J*,*j*=1,...,*J*) entry equal to *ρ*_*i*,*j*_. If a single critical value *c* is used to declare significance, then the probability of not rejecting any null hypothesis is given by

3 $$  \int_{-\infty}^{c}\! \int_{-\infty}^{c}\!...\!\int_{-\infty}^{c} \pi_{Z}((z_{1},z_{2},\dots,z_{J})^{\prime},\boldsymbol{0},\boldsymbol{\Sigma}_{Z})dz_{J} dz_{J-1}\dots dz_{1},  $$

where *π*_*Z*_(***z***,***μ***,***Σ***_*Z*_) is the probability density function of a multi-variate normal (MVN) distribution with mean ***μ*** and covariance matrix ***Σ***_*Z*_.

To control the FWER at level *α*, *c* is chosen such that the integral in Eq. (3) is equal to 1−*α*. Controlling the FWER under the global null in this case ensures strong FWER control.

### Extension of the Dunnett test when a treatment arm is added during a trial: determining the correlation between test statistics

he main difference here to the standard Dunnett test is that the patients from the control arm used for each treatment-to-control comparison no longer completely overlap, and this changes the correlation between the test statistics.

The test statistic of interest at the end of the study, comparing experimental treatment *j* (*j*=1,…,*J*) to control, is given by

4 $$  U_{j} = \frac{\bar{X}_{j}-\bar{X}_{0k_{(j)}}}{\sqrt{\frac{1}{n_{j}}+\frac{1}{\sum\limits_{k\in k_{(j)}} n_{0k}}}} \sim N\left(0,\sigma^{2}\right),  $$

where $\bar {X}_{0k_{(j)}} = \frac {\sum \limits _{k \in k_{(j)}} \sum \limits _{i=1}^{n_{0k}} X_{i0k}}{\sum \limits _{k \in k_{(j)}}n_{0k}}$ and *k*_(*j*)_ denotes the set of stages for which controls are randomised concurrently to treatment *j*. $(U_{1},\dots,U_{J})^{\prime }\sim MVN(\boldsymbol {0},\Sigma _{U})$, where ***0*** is the zero vector of length *J*, *Σ*_*U*_ is the *J*×*J* matrix with *jj*^*th*^ (*j*=1,...,*J*) entry equal to *σ*^2^ and *ij*^*th*^ (*i*≠*j*,*i*=1,...,*J*, *j*=1,...,*J*) entry equal to *σ*^2^*ρ*_*i*,*j*_, with correlation given by

5 $$  {\begin{aligned} \rho_{i,j}=\frac{1}{\sqrt{\frac{1}{n_{1}}+\frac{1}{\sum\limits_{k \in k_{(1)}}n_{0k}}}\sqrt{\frac{1}{n_{2}}+\frac{1}{\sum\limits_{k\in k_{(2)}}n_{0k}}}}\ \frac{\sum\limits_{k\in k_{(1)}\cap k_{(2)}}n_{0k}}{\left(\sum\limits_{k \in k_{(1)}}n_{0k}\right)\left(\sum\limits_{k \in k_{(2)}}n_{0k}\right)}, \end{aligned}}  $$

where $\sum \limits _{k\in k_{(1)}\cap k_{(2)}}n_{0k}$ represents the number of overlapping controls. For equal sample sizes per treatment group, the correlation simplifies to

6 $$  \frac{\sum\limits_{k\in k_{(1)}\cap k_{(2)}}n_{0k}}{2\times \sum\limits_{k \in k_{(1)}}n_{0k}}.  $$

When a treatment arm is added during the trial, only some controls are used in both treatment comparisons, and therefore the correlation between test statistics is reduced.

The correlation is 0.5 when there is complete overlap in the controls for each treatment group and zero when there is no overlap in controls. The derivation of Eq. (5) is given in Additional file 1: Section 1.1. Note that Eq. (2) is a special case of Eq. (5).

As in the “Dunnett test” section, we also consider the standardised test statistics, *Z*_*j*_=*U*_*j*_/*σ*. Under the null hypothesis, $(Z_{1},\dots,Z_{J})^{\prime }\sim MVN(\boldsymbol {0},\Sigma _{Z})$, where ***0*** is the zero vector of length *J*, *Σ*_*Z*_ is the *J*×*J* matrix with *jj*^*th*^ (*j*=1,...,*J*) entry equal to 1 and *ij*^*th*^ (*i*≠*j*,*i*=1,...,*J*,*j*=1,...,*J*) entry equal to *ρ*_*i*,*j*_, given in Eq. (5). If a single critical value *c* is used to declare significance, then the probability of not rejecting any null hypothesis is given by

7 $$  {\int_{-\infty}^{c}\! \int_{-\infty}^{c}\!...\int_{-\infty}^{c}\! \pi_{Z}((z_{1},z_{2},\dots,z_{J})^{\prime},\boldsymbol{0},\boldsymbol{\Sigma}_{Z})dz_{J} dz_{J-1}\dots dz_{1},}  $$

where *π*_*Z*_(***z***,***μ***,***Σ***_*Z*_) is the probability density function of an MVN distribution with mean ***μ*** and covariance matrix ***Σ***_*Z*_.

To control the FWER at level *α*, *c* is chosen such that the integral in Eq. (7) is equal to 1−*α*.

### Adaptive design

The proposed design adapts the trial at the time point when the new treatment arm is added, whilst ensuring the FWER is controlled. It is assumed that the decision to add a new treatment arm to the trial is driven by external reasons. This does not require looking at the current trial data, and therefore the FWER is not inflated due to interim analyses [1]. The aim of the trial is to identify whether any or all treatments are better than control, and it is assumed that only controls randomised concurrently are used in the analysis for each treatment. Here we assume the treatment effect to be detected is the same for all *J* experimental treatments. The null hypotheses are *H*_0_:*μ*_*j*_=*μ*_0_, and the alternative hypotheses are *H*_1_:*μ*_*j*_>*μ*_0_. For illustration we start with a two-arm trial and add an experimental arm, assuming that the experimental treatment arm added during the trial aims to detect the same treatment difference and has the same standard deviation as the original treatment arm. The two main adjustments to the design when adding a treatment are as follows: changing the error control (from pairwise to FWER if going from a two-arm to a multi-arm trial or increasing the size of the "family" for FWER control when adding an additional experimental treatment to a multi-arm trial), which will result in an increase in sample size to maintain the marginal power for each treatment comparison at the same level as before the treatment arm is added; adding more control patients for the new treatment arm comparison since only concurrent controls are used for a given treatment comparison. The effects of the change in error control and the additional controls on the total sample size of a trial where a treatment arm is added are explored further in the Results section.

The sample size required per treatment group for a two-arm trial is calculated using the standard formula

$$ n = \frac{2\sigma^{2}(Z_{1-\alpha}-Z_{\beta})^{2}}{(\mu_{1}-\mu_{0})^{2}},   $$

where *α* is the significance level, *β* is the probability of a type II error and *Z*_1−*α*_ and *Z*_*β*_ are the (1−*α*)th and the *β*th quantiles of the standard normal distribution.

**Fig. 1:**
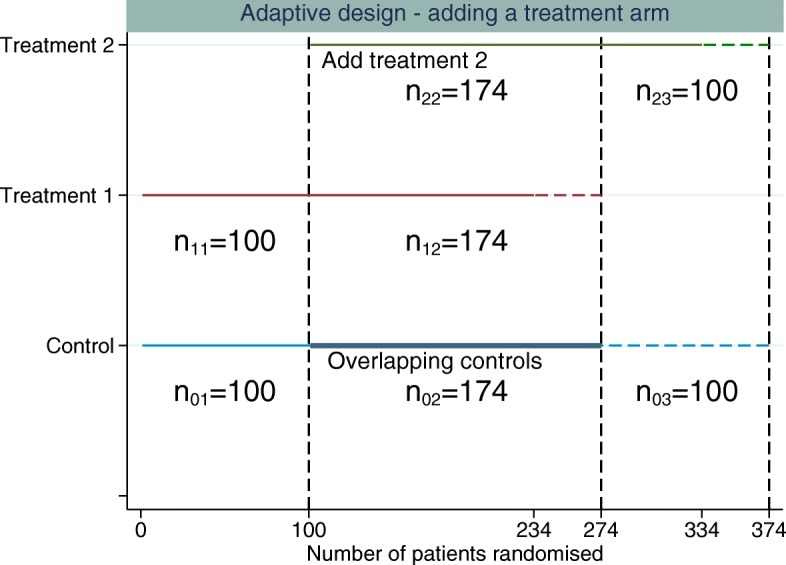
Example of adding a single experimental treatment arm to a two-arm trial comparing treatment 1 to control. The *first dashed vertical line* represents when the new treatment arm (treatment 2) is added to the trial. The *second dashed vertical line* represents when the original treatment (treatment 1) finishes recruitment and the *third dashed vertical line* represents when the control and treatment 2 finish recruiting patients. The *horizontal dashed lines* represent the additional patients required per treatment group above the original sample size estimate to control the FWER whilst maintaining randomisation of 1:1:1 to all treatment arms

The new treatment arm is added after *n*_01_ patients have been randomised to control. The design used for illustration here has three stages (*K*=3). We assume equal randomisation to all treatment arms throughout, which will give an equal number of patients per group for each treatment-to-control comparison over the whole trial. Then *n*_01_+*n*_02_ is the control sample size for the treatment 1 comparison, *n*_02_+*n*_03_ is the control sample size for the treatment 2 comparison, *n*_11_+*n*_12_ is the treatment 1 sample size and *n*_22_+*n*_23_ the treatment 2 sample size, *n*_21_=0, *n*_13_=0, and within each stage, the sample sizes per arm are equal; i.e. the allocation ratio is 1:1 between any treatment arms recruiting in a given stage (as illustrated in Fig. 1).

To maintain the marginal power whilst controlling the FWER at level *α*, the number of patients randomised to every treatment arm is increased when the new treatment arm is added to the trial, using the following steps:

1. Estimate the correlation assuming the original sample size *n* for all treatment groups. The correlation is given by $\rho _{i, j}=\frac {n_{02}}{2n}$.
2. Increase the sample size of all treatment arms to maintain the marginal power for each pairwise comparison whilst controlling the FWER at level *α*. Determine the critical value, *c*, that controls the FWER at level *α* using
  $$ \int_{-\infty}^{c} \int_{-\infty}^{c} \pi_{Z}((z_{1},z_{2})^{\prime},\boldsymbol{0},\boldsymbol{\Sigma}_{Z}) dz_{2} dz_{1} = 1-\alpha $$
  where *π*(***z***,***0***,***Σ***_*Z*_) is the probability density function of an MVN distribution with mean ***0*** and covariance matrix $\boldsymbol {\Sigma }_{Z} = \left (\begin {array}{cc} 1 & \rho _{1, 2} \\ \rho _{2, 1} & 1 \\ \end {array}\right)$.
  The critical value here is determined using MVN integration and a root-finding algorithm in Stata which implements Brent’s method [5].
  The sample size required per group is then given by
  $$n = \frac{2\sigma^{2}(Z_{\Phi(c)}-Z_{\beta})^{2}}{(\mu_{j}-\mu_{0})^{2}} $$
3. Re-estimate the correlation using the sample sizes obtained in step 2, and repeat step 2 using this correlation until the correlation does not change to a small accuracy.

The estimate of the correlation between test statistics at the end of the study using the original sample size calculation will be an underestimate, since, when the sample size of all treatment groups is increased, the number of overlapping controls will increase and therefore the correlation will increase. This is why an iterative approach is required. Based on the final re-calculated sample size, *n*, and a 1:1 allocation ratio in each stage, *n*_02_=*n*_12_=*n*_22_=*n*−*n*_01_ and *n*_03_=*n*_23_=*n*−*n*_02_.

The same methodology applies when adding an additional experimental treatment arm to a multi-arm trial. However, if the original trial was a multi-arm trial, the initial sample size for each treatment arm would have been chosen to control the FWER (e.g. using the method described in the “Dunnett test” section).

### Optimal allocation

In multi-arm trials there are various ways to define the power of a study. The marginal power has been used in this paper so far, which is the probability of rejecting a particular false null hypothesis. How the power is defined for a study depends on the research question that the study is designed to address. In a single trial, where we are comparing multiple experimental treatments to control, the research question could be to determine whether all treatments are better than control, or at least one treatment is better than control. Therefore, the definition of power will depend on the study objectives and assumptions. A comprehensive review of power definitions in multi-arm trials is given in [6]. In this section, we assume that the overall power of the study is of interest. Here, the overall power of a multi-arm trial is defined to be the probability of rejecting all false null hypotheses, correctly determining all treatments that are better than control. The overall power is considered in this section, with the aim of determining the optimal sample size which gives the highest probability of finding all treatments that are better than control. The overall power was used for optimisation, since we assumed that the expected treatment effect in all arms was the same.

For a standard multi-arm trial, [2] showed that the optimal allocation was $n_{0}=n\sqrt {J}$ [2].

#### Treatments finish recruiting simultaneously

The optimal allocation ratio once a new experimental treatment arm is added is calculated by numerically maximising the overall power for a fixed total sample size. The test statistics of interest at the end of the study are the standardised test statistics *Z*_*j*_=*U*_*j*_/*σ*, where *U*_*j*_ is defined in Eq. (4) and *k*_(1)_={1,2} and *k*_(2)_={2}. The optimal allocation ratios are then determined as follows:

1. Follow the steps in the “Adaptive design” section to determine the required total sample size, *N*, when adding a treatment arm during the trial using 1:1:1 allocation to all treatment arms, where 1:1:1 denotes the allocation ratio of control:treatment 1:treatment 2. The total sample size is fixed at *N*. The new treatment arm is added at a fixed time point after *n*_01_=*n*_11_ patients have been randomised to the original treatment and control. It is then determined how best to allocate the remaining patients.
2. Determine the optimal allocation ratios in stage 2 of the trial that maximise the overall power. The estimates of the correlation and critical value from step 1 are used.
  The correlation based on the total sample size calculation in step 1 is given by $\rho _{{1}, {2}}=1/2\left (\frac {n_{01}}{n_{02}}+1\right)$, where *n*_01_ is the number of control patients randomised before the new treatment arm is added, and *n*_02_ is the number of overlapping controls. The remaining patients to be randomised can then be written as *R*=*N*−(*n*_01_+*n*_11_). The total number of patients is given by *N*=*n*_01_+*n*_02_+*n*_11_+*n*_12_+*n*_22_ (there is no stage 3 since it is assumed that randomisation continues to all treatment arms until the end of the study).
  To determine the optimal allocation for the remaining patients once the new treatment arm is added, the randomisation allocation ratio to the new treatment is fixed at 1, and the remaining patients to be randomised at the time point that the new treatment arm is added is written as
  $$ R=\underbrace{\lambda_{02} n_{22}}_{n_{02}} +n_{22}+\underbrace{\lambda_{12} n_{22}}_{n_{12}},   $$
  where *λ*_*jk*_ denotes the allocation ratio for which patients are allocated to treatment *j* in stage *k* in relation to treatment 2 in stage *k*.. The values of *λ*_02_ and *λ*_12_ are determined that maximise the overall power, which is defined by
  8 $$ 1-\beta = \int_{c1^{*}}^{\infty} \int_{c2^{*}}^{\infty} \pi_{Z}((z_{1},z_{2})^{\prime},\boldsymbol{0},\boldsymbol{\Sigma}_{Z})dz_{2} dz_{1},  $$
  where *π*_*Z*_((*z*_1_,*z*_2_)^′^,***0***,***Σ***_*Z*_) is the bivariate normal distribution with mean ***0*** and variance covariance matrix
  $$\boldsymbol{\Sigma}_{Z} = \left(\begin{array}{cc} 1 & \rho_{1, 2} \\ \rho_{2, 1} & 1 \\ \end{array}\right), $$
  where *ρ*_1,2_ is the correlation calculated in step 1, defined in Eq. (6).
  *c*1^∗^ and *c*2^∗^ in Eq. (8) are defined by
  9 $$  c1^{*} = \frac{\left(c\sqrt{\frac{2\sigma^{2}}{n}}\right)-(\mu_{1}-\mu_{0})}{\sqrt{\frac{\sigma^{2}}{n_{01}+n_{02}}+\frac{\sigma^{2}}{n_{11}+n_{12}}}},  $$
  10 $$ c2^{*}= \frac{\left(c\sqrt{\frac{2\sigma^{2}}{n}}\right)-(\mu_{2}-\mu_{0})}{\sqrt{\frac{\sigma^{2}}{n_{02}}+\frac{\sigma^{2}}{n_{22}}}},  $$
  where *c* is the Dunnett critical value and *n* the sample size per comparison group estimated in step 1. (*μ*_*j*_−*μ*_0_) is the treatment effect under the alternative hypothesis, and
  $ n_{22}=\frac {R}{\lambda _{02}+1+\lambda _{12}}$, $ n_{12}=\frac {R}{\lambda _{02}+1+\lambda _{12}}\times \lambda _{12}$, $ n_{02} = \frac {R}{\lambda _{02}+1+\lambda _{12}}\times \lambda _{02} $, where *λ*_02_ and *λ*_12_ are the allocation ratios that maximise the overall power. This change in allocation ratio will alter the correlation. As in the “Adaptive design” section, iteration is required to control the FWER at the desired level.
3. Re-estimate the correlation and critical value that control the FWER based on the sample sizes per comparison group calculated in step 2. The sample sizes per comparison group are now unequal, and Eq. (5) is required to estimate the correlation.
4. Repeat step 2 (replacing the Dunnett critical value and variance in the numerators of Eqs. (9) and (10) with the critical value and variance calculated using the sample sizes that maximise the overall power. Replace the correlation in Eq. (8) with the correlation calculated using the optimal sample sizes), and repeat step 3 until the optimal allocation is determined that maximises the overall power and where the FWER is also controlled at the desired level.
  The FWER is controlled using an iterative approach as in the “Adaptive design” section. For the optimal allocation design, the overall sample size is fixed, and the allocation ratios are determined which maximise the overall power and control the FWER, whereas for the equal allocation design, the sample size is determined which ensures a desired fixed marginal power and controls the FWER.

#### Optimal allocation when treatments finish recruiting at different time points

Rather than recruiting to all treatment arms until the end of the study and reducing the allocation to the original treatment arm, the original treatment arm can finish recruitment early. Reducing allocation to the original experimental treatment arm when the new treatment arm is added delays learning about the effect of the original treatment arm and may affect patient accrual. Patients may be more willing to participate in the trial if they have equal chance of receiving either of the experimental treatments. This design will randomise more controls in stage 2 and fewer in stage 3, which will also increase the overlapping controls and therefore the correlation.

Again, we assume *λ*_*jk*_ denotes the allocation ratio for which patients are allocated to treatment *j* in stage *k* in relation to treatment 2 in stage *k*. The randomisation allocation ratio to the new treatment is fixed at 1, and *λ*_12_ is also fixed at 1, so in stage 2, the allocation to treatment 1 and treatment 2 is equal. *λ*_03_ is also fixed, adding a constraint on the minimum allocation to control in stage 3 of the trial. If *λ*_03_ was not fixed, optimal allocation would always allocate zero patients to control in the third stage and randomise all controls in stage 2. This happens because all controls would then be used for treatment 1 to control comparison. However, some randomised controls are required in stage 3. The number of remaining patients to be randomised, *R*, can then be written as

$$R=\underbrace{\lambda_{02}n_{22}}_{n_{02}}+\underbrace{ n_{22}}_{n_{12}}+n_{22} +n_{23}+\underbrace{\lambda_{03} n_{23}}_{n_{03}}. $$

Optimisation is required to determine the values of *λ*_02_ and *n*_22_ or *n*_23_ that maximise the overall power. Only one of *n*_22_ or *n*_23_ is required since *N*, *n*_01_ and *n*_11_ are all fixed values. More details are given in Additional file 1: Section 1.2.

## Results

Here we assume an initial two-arm confirmatory randomised controlled trial is designed to detect a treatment difference of 3 between the control and the experimental treatments. The standard deviation is assumed to be 10 in both the experimental treatment and the control arms, and the allocation is 1:1. It is assumed that the experimental treatment arm added during the trial aims to detect the same treatment difference and has the same standard deviation. The outcome data are assumed to be normally distributed and the standard deviation is assumed to be known for all treatment arms. The new treatment arm is added based on external reasons. The aim of the trial is to identify whether any or all experimental treatments are better than control, and only controls randomised concurrently are used for each treatment comparison.

### Two experimental treatments: independent trials

In this section, trials comparing multiple experimental treatments to control in a single trial are compared to running separate trials, each with an independent control arm. For separate, independent trials, the standard approach is to control the marginal type I error rate of each study. In a single study with multiple treatment arms, the aim is to control the FWER.

For an individual trial, comparing one experimental treatment to control, 234 patients are required per treatment group for a one-sided test at level *α*=0.025 and 90% power. Therefore, for two independent trials, the total sample size required is *N*=2*Jn*=936 patients, where *n*=234, the number of patients in each treatment arm. The critical value for each test is approximately 1.96. For two independent trials, the FWER is 1−(1−0.025)^2^=0.0494. For independent trials, the number of type I errors is a binomial random variable with *J* trials and probability of success *α* [7]. To control the FWER at 2.5% when conducting individual trials, 276 patients are required per treatment group, giving a total sample size of *N*=2*Jn*=1104.

### Three-arm trial design: no multiplicity correction

To assess two experimental treatments in a single trial, there are two experimental arms and a single control arm. Making no adjustment for multiplicity, the total number of patients required is *N*=(*J*+1)*n*=702.

The probability of rejecting at least one null hypothesis (FWER) here is given by

$${\begin{aligned} = 1-\left(\int_{-\infty}^{\Phi^{-1}(1-0.025)} \int_{-\infty}^{\Phi^{-1}(1-0.025)} \pi_{Z}((z_{1},z_{2})^{\prime},\boldsymbol{0},\boldsymbol{\Sigma}_{Z})dz_{1} dz_{2}\right) = 0.0454, \end{aligned}} $$

where *Z*_1_ and *Z*_2_ are both ∼*N*(0,1) and we assume equal sample sizes per group and $\boldsymbol {\Sigma }_{Z} = \left (\begin {array}{cc} 1 & 0.5 \\ 0.5 & 1 \\ \end {array}\right)$.

### Three-arm trial design: Dunnett multiplicity correction

This section explores a standard multi-arm trial comparing two experimental treatments, that is *J*=2, to a control treatment. All treatment arms recruit from the beginning of the study and stop recruiting simultaneously, that is *K*=1, so that Dunnett’s method is used to adjust for multiple testing.

To control the FWER at 2.5% and the marginal power of each comparison at 90%, 272 patients are required per treatment group for a one-sided test, *n*=*n*_01_=*n*_11_=*n*_21_=272 (3 groups = 816 total).

The critical value for each individual test is now increased to 2.21 to control the FWER at 2.5%. In the “Two experimental treatments: independent trials” and “Three-arm trial design: no multiplicity correction” sections, a standard normal 2.5% critical value of *Φ*^−1^(1−0.025)≈1.96 was used.

### Adding an arm during the trial: no multiplicity correction

A trial starts with two treatment arms initially, and the new treatment arm is added after 100 patients have been randomised to each treatment group. It is assumed that no correction is made to the critical value for multiple comparisons (the critical value of ≈1.96 is used for both treatment comparisons at the end of the study). A total of 234 patients are allocated to the new treatment and a further 100 patients are randomised to control. The numbers of concurrent controls randomised for each treatment arm are then equal. The number of controls used in both treatment comparisons is 134. The total number of patients per treatment group for each of the treatment comparisons, *n*, is 234 (134 of the controls are used in both treatment comparisons (overlapping controls)). Therefore, *n*_01_=*n*_11_=100, *n*_02_=*n*_12_=*n*_22_=134, *n*_03_=*n*_23_=100 and *n*_13_=0 (*n*=*n*_01_+*n*_02_=*n*_11_+*n*_12_=*n*_22_+*n*_23_=*n*_02_+*n*_03_). The correlation is 0.286 from Eq. (6), and the FWER is 0.0477. The total number of patients required for this design is 802 (3×234+100 extra controls).

The FWER is less for this design (0.0477) than the FWER obtained from two separate trials (0.0494) and requires 134 fewer controls than two separate trials. However, some may argue that, for the single trial, the FWER should be controlled at the 2.5% *α* level of a single independent trial. This design is considered in the next section.

### Adding an arm during the trial: Dunnett multiplicity correction

Figure 1 illustrates the design proposed in the “Adaptive design” section. The estimate of the correlation using the original sample size of 234 per group is 0.286. Using this correlation, the critical value that controls the FWER at 0.025 is 2.2295, requiring a sample size per group of 273.9. Re-estimating the correlation until it does not change much per iteration gives a final correlation of 0.317, a critical value of 2.2277 and a sample size per group of 273.7. Since we require an integer sample size, the change in correlation has no impact on the required sample size for this design. The increase in the number patients required comes from maintaining the marginal power for each pairwise comparison whilst controlling the FWER because the treatment comparisons are within the same trial rather than separate trials. The total sample size required per group (*n*) to control the FWER at 2.5% based on this correlation estimate would be 274 (an additional 40 patients per group). Three groups of 274 patients plus 100 extra non-overlapping controls gives a total sample size of *N* = 922, just below the 936 patients required to run two separate trials. However, here we are controlling the FWER at 2.5%, whereas for two separate studies, only the marginal type I error rate for each comparison is controlled at 2.5%. For this design, *n*=*n*_01_+*n*_02_=*n*_11_+*n*_12_=*n*_22_+*n*_23_=*n*_02_+*n*_03_.

Table 1 compares the FWER, critical value and the number of patients required to have 90% marginal power for each treatment-to-control comparison for the example and designs discussed in the “Two experimental treatments: independent trials” through “Adding an arm during the trial: Dunnett multiplicity correction” sections. From Table 1, for this example, in terms of the number of patients required, a single trial design adding a treatment arm requires fewer patients than a separate trial design, even when controlling the FWER at the level of the type I error rate for a two-arm trial.

**Table 1:** FWER, sample size, critical value (CV) and overall power comparisons for 90% marginal power

Design	FWER	Total sample size	CV	Overall power
Two separate trials	0.0494	936	1.96	0.81
Two separate trials: adjustment for multiplicity	0.0250	1104	2.24	0.81
Single trial: no multiplicity adjustment	0.0454	702	1.96	0.83
Single trial: Dunnett adjustment for multiplicity	0.0250	816	2.21	0.83
Adding an arm: no multiplicity adjustment	0.0477	802	1.96	0.82
Adding an arm: Dunnett adjustment for multiplicity	0.0250	922	2.23	0.82

An example of adding two treatment arms during the trial and using the adaptive design proposed to control the FWER is given in Additional file 1: Section 1.3.

### Comparison of a single trial adding a treatment arm to independent trials

**Fig. 2:**
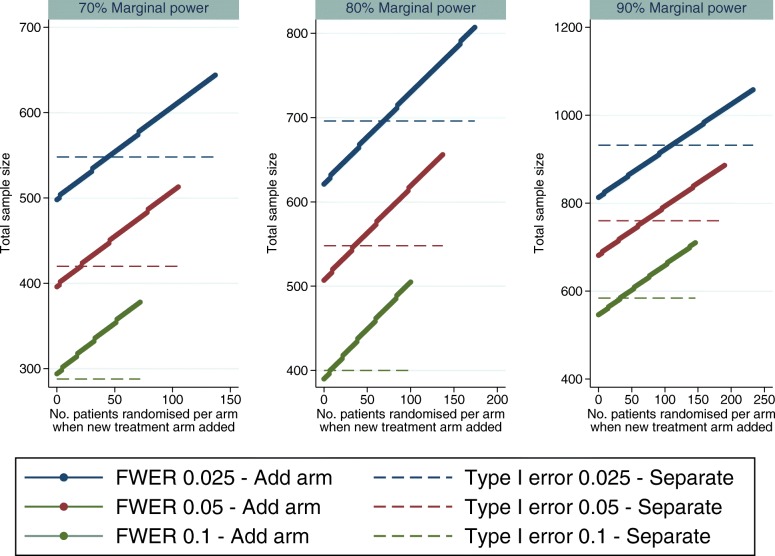
Comparing a single trial to separate trials when a treatment arm is added at different time points during the trial, for varying power (70%, 80% and 90%) and FWER (2.5%, 5% and 10%). It is assumed here that randomisation is 1:1:1 and the treatment effect to be detected is the same for all treatments.

When adding a treatment arm to a trial, the total sample size is increased to maintain the marginal power for each pairwise comparison whilst controlling the FWER and to provide concurrent controls for the new experimental treatment. If control of the FWER is required in a single study and not in separate trials, it is of interest to determine the time point at which it is better to start a separate trial rather than add an arm to an ongoing trial with respect to the total sample size required. This is comparing a single trial controlling the FWER at 2.5% (as described in the “Adding an arm during the trial: Dunnett multiplicity correction” section) to separate trials (the independent trials design described in the “Two experimental treatments: independent trials” section), each with a 2.5% type I error rate. This is illustrated in Fig. 2 for varying marginal power, time points and error rates.

In Fig. 2, the time point where the lines cross shows when it would be better to run a new trial than add a new treatment to the current trial, in terms of the number of patients required, if in a single trial control of the FWER is required at the 2.5% level. The statistical advantage of adding a treatment arm and running a single trial decreases as the power decreases and FWER increases.

### Optimality

**Fig. 3:**
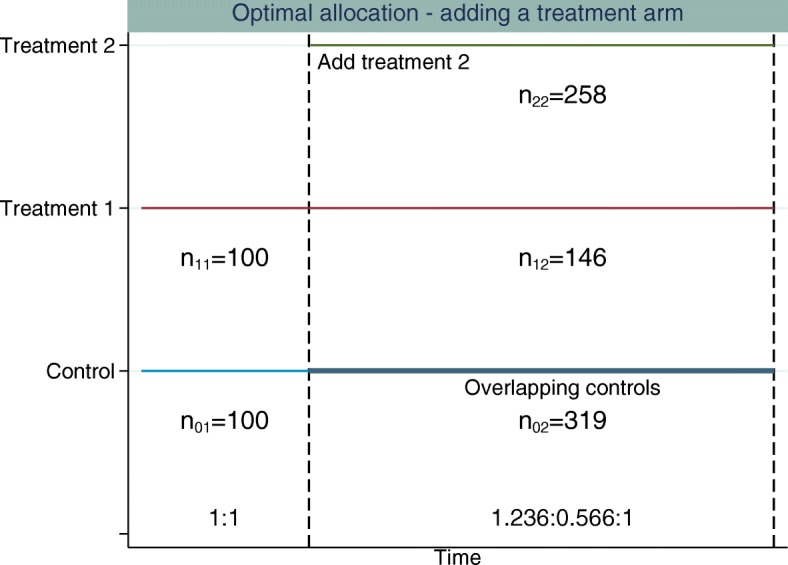
Example of adding a single experimental treatment arm to a two-arm trial comparing treatment 1 to control. The *first dashed vertical line* represents when the new treatment arm (treatment 2) is added to the trial. The *second dashed vertical line* represents when all treatments finish recruiting. The allocation ratio is adapted when treatment 2 is added and all treatments finish recruiting simultaneously. The allocation ratios and sample sizes in each stage for each treatment are displayed

**Fig. 4:**
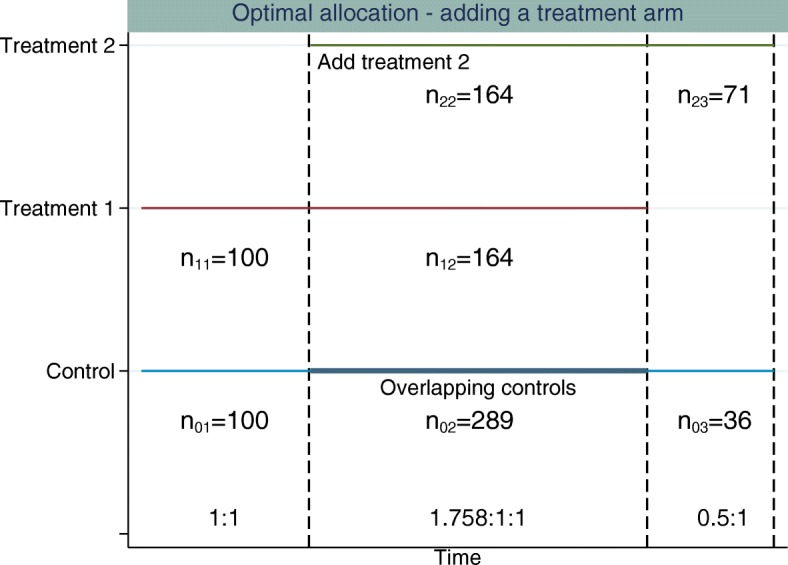
Example of adding a single experimental treatment arm to a two-arm trial comparing treatment 1 to control. The *first dashed vertical line* represents when the new treatment arm (treatment 2) is added to the trial. The *second dashed vertical line* represents when treatment 1 finishes recruiting and the *third dashed vertical line* represents when treatment 2 and control finish recruiting. The allocation ratios are adapted when treatment 2 is added to the trial and again when treatment 1 finishes recruiting. The allocation ratios and sample sizes in each stage for each treatment are displayed

Figures 3 and 4 illustrate the optimal allocation ratios when all treatment arms finish recruiting simultaneously and when the original experimental treatment arm finishes recruitment early, respectively. Up to the time point that the new treatment arm is added, the optimal allocation is 1:1, treatment to control. The total sample size is fixed at 922 patients (274 patients per group plus 100 extra controls). This is the sample size required to obtain 90% marginal power for both treatment comparisons and control the FWER at 2.5% when using 1:1:1 allocation. This design has an overall power of 0.822.

Using optimal allocation, the overall power when treatments finish simultaneously is 86.24% and the marginal power is 91.23% for the new treatment-to-control comparison and 93.43% for the original treatment-to-control comparison. When the original treatment finishes recruitment early, if we fix the treatment 2 to control allocation in stage 3 to be 1:0.5, treatment to control, the overall power is 85.20%. The marginal power is 89.83% for the new treatment-to-control comparison and 93.74% for the original treatment-to-control comparison.

All of the optimal allocation methods considered achieve higher overall power compared to an equal allocation (where equal allocation is 1:1 or 1:1:1 allocation across all treatment arms recruiting within stage *k*, the overall power for equal allocation is 82.2%) design for the same sample size. The power calculations are based on the unrounded optimal sample size estimates. The sample sizes are then rounded for each stage, which may affect the operating characteristics slightly.

Table 2 compares the overall power for different fixed stage 3 allocation ratios, and Fig. 4 illustrates the design for the stage 3 fixed allocation ratio of 1:0.5, treatment to control. The marginal power for the treatment 2-to-control comparison can be less than the marginal power obtained when using 1:1:1 allocation under this design where the allocation ratios are adapted and the original experimental treatment can finish recruitment before the new experimental treatment arm finishes recruitment, since the optimal allocation is determined that maximises the overall power. The gain in power from the treatment 1-to-control comparison can outweigh that of the treatment 2-to-control comparison. Furthermore, placing a higher constraint on the number of controls that are to be randomised in stage 3 of the trial results in fewer patients being randomised to both treatment 2 and control in stage 3 and a larger number of patients being randomised in stage 2.

**Table 2:** Allocation ratios, overall power and sample size comparisons for the adding a treatment arm design using optimal allocation when treatments finish recruitment at different times

Stage 3 ratio	Stage 2 ratio	*n*_23_	Correlation	Overall power
1:0.2	2.015:1:1	100	0.170	0.852
1:0.3	1.932:1:1	93	0.176	0.850
1:0.4	1.856:1:1	85	0.181	0.848
1:0.5	1.791:1:1	78	0.186	0.847
1:0.6	1.730:1:1	71	0.191	0.845
1:0.7	1.678:1:1	64	0.197	0.844
1:0.8	1.632:1:1	58	0.202	0.843

The total sample size of the trial is based on an adding a treatment arm with 1:1:1 allocation design with a marginal power of 90% and an FWER of 2.5%

## Discussion

In this paper we propose a design for adding a treatment arm to an ongoing trial which controls the FWER. We then explore changing the allocation ratio to each treatment arm at the point when the new treatment arm is added to increase the overall power of the trial compared to 1:1:1 randomisation. The methods discussed here are illustrated for adding a single treatment arm to an ongoing trial, but they can easily be used for adding multiple treatment arms, as illustrated in Additional file 1: Section 1.3.

An increase in the sample size of all treatment arms is required when adding a treatment arm to a trial if it is desired to maintain the marginal power for each pairwise comparison whilst control of the FWER is required at the level of the type I error in the original study. The change in correlation makes little difference to the increase in sample size, but iteration is required to guarantee control of the FWER in a single trial when adding a treatment arm. When control of the FWER is required in a single trial which adds an additional experimental treatment arm, but control of the FWER is not required in separate trials, in terms of the number of patients required and depending on the time point at which the treatment arm is added and the power and error required for the trial, it may be better to run a separate trial for each experimental treatment.

Whether control of the FWER is required under these assumptions has been widely discussed [7–12]. The main argument for not adjusting for multiplicity is that if the same two comparisons were made in separate trials, controlling the FWER would not be required. The use of the same control group in both treatment comparisons in a multi-arm trial is an argument for having a larger control group in a multi-arm trial. It has been considered that due to random chance the control group in a multi-arm trial could overestimate or underestimate the true treatment effect. This control group is then used for all treatment-to-control comparisons [7]. An increase in the sample size of the control arm will reduce some of the sample variation in the controls. Since the regulatory advice is to control the FWER in this setting, this was the approach considered in this paper. However, it is important to emphasise that if we compared designs like for like in terms of FWER (i.e. controlling the FWER when running a separate trial for each experimental treatment as well as when comparing multiple experimental treatments to control in a single trial), adding an arm partway through the trial is better than running two separate trials in terms of sample size, time and logistics.

A further consideration for the adaptive design proposed is that whilst it is beneficial to be able to re-calculate the sample size of the trial at the point when the arm is added, it may not be appealing to sponsors at the design stage of the trial not to know how many patients may be required. Knowing that it is planned to add a treatment arm at some point during the trial, at the design stage of the original trial, the total sample size required for adding a treatment arm using the adaptive design can be calculated for adding a new treatment arm at different time points, as illustrated in the “Comparison of a single trial adding a treatment arm to independent trials” section. A maximum sample size can then be calculated at the design stage which ensures control of the FWER, and decisions can be made as to whether a single study adding a treatment arm or independent trials are required based on both statistical and logistic factors.

To determine the optimal allocation ratio, it was assumed that the total sample size of the study was fixed. Based on this total sample size, the optimal allocation for the remaining patients to be randomised after the treatment arm was added was determined. The allocation ratio was determined that maximised the probability of detecting a treatment effect in both arms. This was chosen because we assumed that the expected treatment effect in all experimental arms was the same. In this case, the marginal power will either increase or be similar to the marginal power in the design assuming equal allocation to all treatment arms. The marginal power may be lower than desired if the overall power is used for optimisation of the allocation ratio and the treatment effects are assumed to differ in each of the experimental arms. Where the treatment effects are assumed to differ, optimisation can be based on maximising the probability of there being no type I errors, as in the original Dunnett paper [2], or the probability that there is a treatment effect in at least one of the treatment arms. The methods described in the “Optimal allocation” section can be used in both of these situations.

In this paper, two definitions of power in multi-arm trials have been considered: detecting a particular effective treatment compared to control, the marginal power, and the overall power, which is the probability of rejecting all false null hypotheses. The definition of power will depend on the study objective, i.e. whether that is to determine all treatments better than control, any treatment better than control or the best treatment. Possible future work will look at designs where a treatment arm is added and the aim is to compare experimental treatments to each other as well as to the control group, in terms of controlling the FWER and optimal allocation.

Current practice in clinical trials that have added a treatment arm is to use only concurrently randomised controls for each treatment-to-control comparison [1]. One reason for this is to preserve randomisation. Incorporating control data from the first stage in the second treatment control comparison is utilising non-randomised information in that comparison. A second reason is that the patient population may differ before and after the treatment arm has been added. Incorporating the first stage control data into the second treatment comparison could then bias the treatment effect estimate for treatment 2. Depending on the possible types of change that occur when a new treatment arm is added, this may affect the original treatment-to-control comparison. However, the use of only concurrent controls guards against some possible biases or loss of power that could occur from adding a treatment arm to an ongoing study.

Possible reasons for a change in the patient population when the new treatment arm is added are the following: a change in patient characteristics, patients may be more willing to be randomised with the possibility of receiving two experimental treatments; a change in baseline characteristics over time; and the possibility that clinicians may be more willing to randomise higher or lower risk patients into the trial because of their expectations of the new treatment. For the designs considered in this paper, it was assumed that the patient population is homogeneous across stages. If it is thought that adding a treatment during the trial may alter the trial in some way, causing a stage effect, adjustment for stage or treatment by stage interactions may be required using a linear regression approach. This method is described in [13] and may reduce the power of the study. The effect of different stage effects was not explored here but was considered in [13].

Throughout this paper, the final analysis assumed a *z* test for each treatment comparison, pooling data across stages but only using concurrent controls and assuming that the population variance is known. In reality, the population variance would not be known; however, for large samples as are likely in a confirmatory trial, the *z* test is adequate. However, if the approximation of the standard deviation was inaccurate, this will affect the operating characteristics of the trial. An approach to handle unknown variance could be adapted from the methods discussed in [14]. Furthermore, only normally distributed outcomes have been considered in this paper. Future work will look to generalise the adaptive design proposed for binary and time-to-event outcomes.

## Supplementary information

**Additional file 1** Supplementary information.

## Data Availability

All data generated or analysed in this paper are included in this published article.
