# Supplementary material for: Designs for adding a treatment arm to an ongoing clinical trial
Source: Trials. 2020 Mar 6;21:251. doi: 10.1186/s13063-020-4073-1 (PMC7060622; doi:10.1186/s13063-020-4073-1)
Supplement: Supplementary file 1 — Additional file 1 Supplementary information. [file 13063_2020_4073_MOESM1_ESM.pdf]

1 Supplementary information

1.1 Derivation of the correlation between test statistics when adding a treatment arm

$$\rho_{1,2} = \frac{Cov\left(\frac{\bar{X}_1 - \bar{X}_{0k_{(1)}}}{\sqrt{\frac{1}{n_1} + \frac{1}{\sum\limits_{k \in k_{(1)}} n_{0k}}}}, \frac{\bar{X}_2 - \bar{X}_{0k_{(2)}}}{\sqrt{\frac{1}{n_2} + \frac{1}{\sum\limits_{k \in k_{(2)}} n_{0k}}}}\right)}{\sigma^2}.$$

Let $\kappa = \frac{1}{\sqrt{\frac{1}{n_1} + \frac{1}{\sum\limits_{k \in k_{(1)}} n_{0k}}}\sqrt{\frac{1}{n_2} + \frac{1}{\sum\limits_{k \in k_{(2)}} n_{0k}}}}$. Then,

$$Cov\left(\frac{\bar{X}_1-\bar{X}_{0k_{(1)}}}{\sqrt{\frac{1}{n_1}+\frac{1}{\sum\limits_{k\in k_{(1)}} n_{0k}}}},\frac{\bar{X}_2-\bar{X}_{0k_{(2)}}}{\sqrt{\frac{1}{n_2}+\frac{1}{\sum\limits_{k\in k_{(2)}} n_{0k}}}}\right)=\kappa\ Cov(\bar{X}_{0k_{(1)}},\bar{X}_{0k_{(2)}})$$

$$=\kappa\ Cov\left(\frac{\sum\limits_{k\in k_{(1)}}\sum\limits_{l=1}^{n_{0k}}X_{l0k}}{\sum\limits_{k\in k_{(1)}} n_{0k}},\frac{\sum\limits_{k\in k_{(2)}}\sum\limits_{m=1}^{n_{0k}}X_{m0k}}{\sum\limits_{k\in k_{(2)}} n_{0k}}\right)$$

$$=\kappa\ \frac{1}{\sum\limits_{k\in k_{(1)}} n_{0k}\sum\limits_{k\in k_{(2)}} n_{0k}}\left(\sum_{k\in k_{(1)}}\sum_{l=1}^{n_{0k}}\right)\left(\sum_{k\in k_{(2)}}\sum_{m=1}^{n_{0k}}\right)\underbrace{\overbrace{Cov(X_{l0k},X_{m0k})}}_{\mathbf{E}(X_{l0k}X_{m0k})-\mathbf{E}(X_{l0k})\mathbf{E}(X_{m0k})}$$

$(k\notin k_{(1)}\cap k_{(2)}\implies \mathbf{E}(X_{l0k}X_{m0k})=\mathbf{E}(X_{l0k})\mathbf{E}(X_{m0k}))$

$$=\kappa\ \frac{1}{\sum\limits_{k\in k_{(1)}} n_{0k}\sum\limits_{k\in k_{(2)}} n_{0k}}\sum_{m\in k_{(1)}\cap k_{(2)}}\sum_{m=1}^{n_{0k}}\underbrace{\mathbf{E}(X_{m0k}X_{m0k})-\mathbf{E}(X_{m0k})\mathbf{E}(X_{m0k})}_{=\mathrm{Var}(X_{m0k})=\sigma^2}$$

(Since $k\in k_{(1)}\cap k_{(2)}\implies X_{l0k}=X_{m0k}$)

$$=\kappa\sigma^2\frac{\sum\limits_{k\in k_{(1)}\cap k_{(2)}} n_{0k}}{\sum\limits_{k\in k_{(1)}} n_{0k}\sum\limits_{k\in k_{(2)}} n_{0k}}.$$

$$\rho_{1,2}=\frac{\frac{1}{\sqrt{\frac{1}{n_1}+\frac{1}{\sum\limits_{k\in k_{(1)}} n_{0k}}}}\frac{1}{\sqrt{\frac{1}{n_2}+\frac{1}{\sum\limits_{k\in k_{(2)}} n_{0k}}}}\frac{\sum\limits_{k\in k_{(1)}\cap k_{(2)}} n_{0k}}{\sum\limits_{k\in k_{(1)}} n_{0k}\sum\limits_{k\in k_{(2)}} n_{0k}}\times\sigma^2}{\sigma^2}$$

$$=\frac{1}{\sqrt{\frac{1}{n_1}+\frac{1}{\sum\limits_{k\in k_{(1)}} n_{0k}}}}\frac{1}{\sqrt{\frac{1}{n_2}+\frac{1}{\sum\limits_{k\in k_{(2)}} n_{0k}}}}\frac{\sum\limits_{k\in k_{(1)}\cap k_{(2)}} n_{0k}}{\sum\limits_{k\in k_{(1)}} n_{0k}\sum\limits_{k\in k_{(2)}} n_{0k}}.$$

This simplifies if the number of patients in each treatment group are equal,

$$\sum_{k \in k_{(1)}} n_{0k} = \sum_{k \in k_{(2)}} n_{0k} = n_1 = n_2 = n \implies \rho_{Z_1,Z_2} = \frac{\sum\limits_{k \in k_{(1)} \cap k_{(2)}} n_{0k}}{2n}.$$

1.2 Optimal allocation algorithm when treatments finish recruiting at different time points

The test statistics here are the same as those given in Equation (4). However, there are now three stages to the design, $k_{(1)} = \{1, 2\}$ and $k_{(2)} = \{2, 3\}$. When all treatments finish recruiting simultaneously, there are only two stages.

1. The total sample size is fixed at N, the number of patients required to control the FWER at level α using equal randomisation when adding a treatment arm during the trial, described in Section 2.4.

   It is then determined how best to allocate the remaining patients, $R = N-(n_{01}-n_{11})$, once the new treatment arm has been added to optimise the overall power. The new treatment arm is added at a fixed time point after $n_{01} = n_{11}$ patients have been randomised to the original treatment and control.

2. Determine the optimal allocation ratios that maximise the overall power. The estimate of the correlation and critical value from step one are used.

   Fixing $\lambda_{12} = \lambda_{22} = \lambda_{23} = 1$, the remaining patients to be randomised, R, can be written as,

$$R = \underbrace{\lambda_{02} n_{22}}_{n_{02}} + \underbrace{n_{22}}_{n_{12}} + n_{22} + n_{23} + \underbrace{\lambda_{03} n_{23}}_{n_{03}}.$$

   Optimisation is required to determine the values of $\lambda_{02}, \lambda_{03}$ and n_{22} or n_{23} that maximise the overall power. Only one of n_{22} or n_{23} are required since N, n_{01} and n_{11} are all fixed values. However, numerical optimisation of these parameters will always allocate zero patients to control in the third stage and randomise all to control in stage two. This is because all controls will then be used for the treatment 1 to control comparison. However, some randomised controls are required in stage three. Therefore, λ_{03} is also fixed, adding a constraint on the minimum allocation to control in stage three of the trial. Numerical

optimisation is then used to find the values of λ_{02} and n_{23} that maximise the overall power. The number of controls available for the first treatment comparison will vary depending on when the original treatment arm finishes recruiting.

The overall power is defined by,

$$1-\beta=\int_{c1^*}^{\infty}\int_{c2^*}^{\infty}\pi_Z((z_1,z_2)',\mathbf{0},\mathbf{\Sigma}_Z)dz_2dz_1, \tag{1}$$

where $\pi_Z((z_1,z_2)',\mathbf{0},\mathbf{\Sigma}_Z)$ is the bivariate normal distribution with mean $\mathbf{0}$ and variance covariance matrix,

$$\mathbf{\Sigma}=\begin{pmatrix}1 & \rho_{1,2}\\ \rho_{2,1} & 1\end{pmatrix},$$

where $\rho_{1,2}$ is the correlation calculated in step one, defined in Equation (6).

$c1^*$ and $c2^*$ in Equation (11) are defined by,

$$c1^*=\frac{\left(c\sqrt{\dfrac{2\sigma^2}{n}}\right)-(\mu_1-\mu_0)}{\sqrt{\dfrac{1}{n_1}+\dfrac{1}{\sum\limits_{k\in k_{(1)}} n_{0k}}}}, \tag{2}$$

and,

$$c2^*=\frac{\left(c\sqrt{\dfrac{2\sigma^2}{n}}\right)-(\mu_2-\mu_0)}{\sqrt{\dfrac{1}{n_2}+\dfrac{1}{\sum\limits_{k\in k_{(2)}} n_{0k}}}}, \tag{3}$$

where $k=1,\ldots,3$, c is the Dunnett critical value and n the sample size per comparison group estimated in step 1. $(\mu_j-\mu_0)$ is the treatment effect under the alternative hypothesis and,

$$n_{22} = \frac{R - (\lambda_{03}+1)n_{23}}{\lambda_{02}+2}, \; n_1 = n_{11} + n_{12}, \; \sum_{k \in k_{(1)}} n_{0k} = n_{01} + n_{22}\lambda_{02}, \; n_2 = n_{22} + n_{23},$$
$$\sum_{k \in k_{(2)}} n_{0k} = n_{22}\lambda_{02} + n_{23}\lambda_{03}.$$

where λ_{03} is fixed and n_{23} and λ_{02} are found via optimisation. This change in allocation ratio will alter the correlation. As in Section 2.4, iteration is required to control the FWER at the desired level.

3. Re-estimate the correlation and critical value that control the FWER based on the sample sizes per comparison group, calculated in step two. The sample sizes per comparison group are now unequal and Equation (5) is required to estimate the correlation.

4. Repeat step 2 (replacing the Dunnett critical value and variance in the numerator of Equations (12) and (13) with the critical value and variance calculated using the sample sizes that maximise the overall power. Replace the correlation in Equation (11) with the correlation calculated using the optimal sample sizes) and repeat step 3 until the optimal allocation is determined that maximises the overall power and where the FWER is also controlled at the desired level.

1.3 Example of adding two treatment arms using the adaptive design with the Dunnett correction

So far, adding only one new treatment arm has been considered. We now consider adding two treatment arms during the trial, correcting for multiple comparisons using the Dunnett procedure. There are now three comparisons of interest, comparing three experimental treatments to control. The correlation between test statistics is given in Equation (5). The three test statistics follow a J-variate normal distribution.

It is assumed that the second experimental treatment arm is added before the original experimental treatment arm finishes recruitment and therefore there is correlation between all test statistics. However, the correlations between all test statistics can never

be equal.

Continuing the main example used throughout this paper, two new treatment arms are added, the first after 200 patients have been randomised to either the original experimental treatment or control with 1:1 allocation.

The variance-covariance matrix using the original sample size calculation of 234 patients per group is given by,

$$\mathbf{\Sigma} = \begin{pmatrix} 1 & 0.286 & 0.073 \\ 0.286 & 1 & 0.286 \\ 0.073 & 0.286 & 1 \end{pmatrix}$$

Based on the above correlation matrix, the sample size required per group to control the FWER at 2.5% and the correlation are re-estimated until they converge. The final sample size required per group is then 298 patients and the correlation matrix is given by,

$$\mathbf{\Sigma} = \begin{pmatrix} 1 & 0.332 & 0.164 \\ 0.332 & 1 & 0.332 \\ 0.164 & 0.332 & 1 \end{pmatrix}$$

Figure 1 illustrates a design where two treatment arms are added. This design has balanced overlap in the number of controls used between treatment 1 and treatment 2 and between treatment 2 and treatment 3. The sample size of all groups is increased to control the FWER and maintain the power for each pairwise treatment comparison using the Dunnett correction. Treatment 2 is added after 200 patients have been randomised to either control or treatment 1. Treatment 3 is then added after a further 100 patients have been randomised to each of the groups. The sample size for each group is increased by 64 patients from 234 to 298, shown in the Figure by the horizontal dashed lines. The

dashed vertical lines show where new treatment arms are added and treatments finish recruiting. The number of overlapping controls now differs between test statistics. Between Z_1 and Z_2, and Z_3 and Z_2 the number of overlapping controls is 198 (as shown by the navy and pink lines) and between Z_3, Z_1 is 98 controls (as shown by the yellow line).

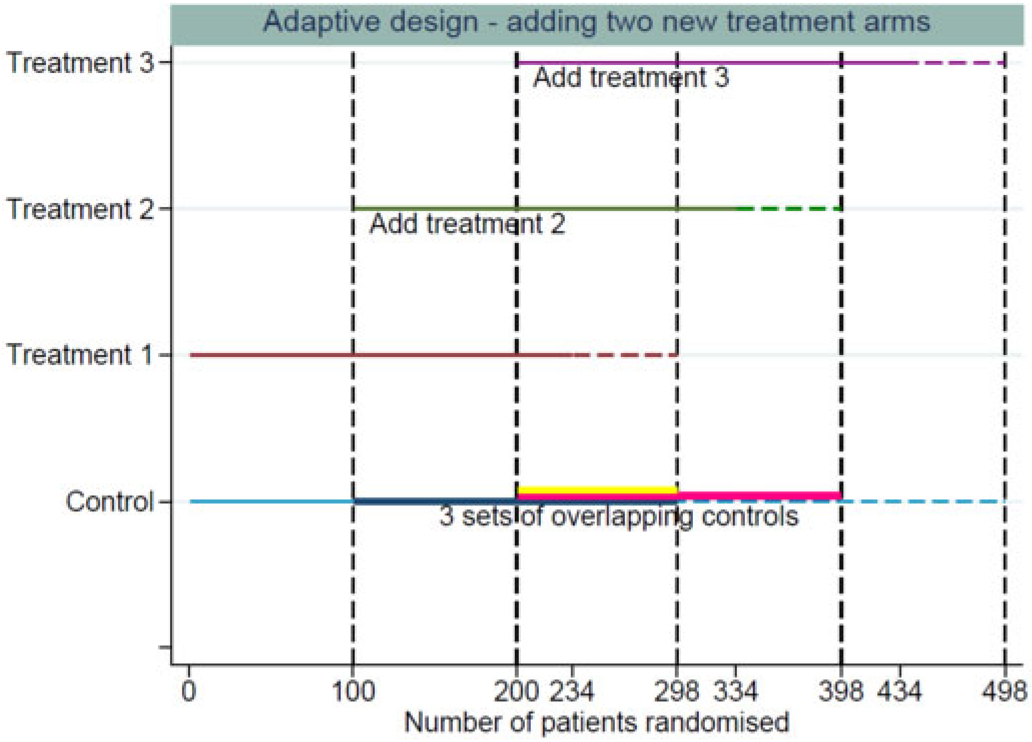

Figure 1: Example of adding two experimental treatment arms (treatment 2 and treatment 3) to a two-arm trial comparing treatment 1 to control. The first dashed vertical line represents when the first new treatment arm (treatment 2) is added to the trial. The second dashed vertical line represents when the second new treatment arm (treatment 3) is added to the trial. The remaining vertical lines are when treatments finish recruiting. The horizontal dashed lines represent the additional patients required per treatment group above the original sample size estimate to control the FWER while maintaining randomisation of 1:1:1 to all treatment arms.

The total sample size required here is 1392 compared to the total sample size of 1404 when running three separate studies. However, this is under control of the FWER at the 2.5% level, the same as the marginal type I error rate for a single study comparing one experimental treatment to control. If this strict control of the FWER is required in a single study compared to running separate trials. The benefits of adding treatment arms to a trial will decrease as the number of treatment arms added increases, if multiple test correction is required for the multi-arm trial but not the separate trials.

So far, only equal allocation to all treatment arms has been considered and power in terms of controlling the marginal power for each treatment to control comparison. We now look at optimal allocation to maximise the overall power of the study.
